# Child and Adolescent Mental Health Services in Oman

**DOI:** 10.1080/17571472.2018.1482661

**Published:** 2018-06-08

**Authors:** Hassan Mirza

**Affiliations:** a Department of Behavioural Medicine, Sultan Qaboos University Hospital, Muscat, Oman

**Keywords:** Adolescent psychiatry, Oman, health resources

## Abstract

Oman is an emerging economy which has witnessed rapid development and demographic shift over the past four decades. With such changes, the country has witnessed a surge in the number of young people with various mental health problems. Despite the increasing need for mental health services for the young, there is a scarcity of such services in Oman due to severe shortage and maldistribution of facilities. Currently, only two institutes in Oman offer child and adolescent mental health services to the whole country. This paper outlines how Oman is delivering mental health services for the young despite the challenges.

## Why this matters to me?

How Oman is faring in delivering mental health services for the young.

## Key message

Child and adolescent mental health professionals in Oman are on a path to success.

The Sultanate of Oman is a country in the Middle East with a population of over 4.5 million [1]. Omani citizens constitute 54% of the population, with half of that population less than 25 years of age [2]. The capital Muscat is the largest city of Oman where the majority of the population resides, and the only city with Child and Adolescent Mental Health Services (CAMHS). Overall, there is a scarcity of CAMHS in Oman.

Oman’s national health system is offered free of charge to all the citizens from birth to grave [3]. The World Health Organization (WHO), in its first-ever comparative analysis of health systems in 2000, ranked Oman first among 191 WHO member states for its overall performance on the level of health [4]. The United Nations 2010 Human Development Report listed Oman at the top of the world’s 10 leading countries that have made the greatest progress in recent decades in public health [5].

As a relatively high-income country in the past four decades, Oman has benefited from improved standards of living, but the country has witnessed a surge in the number of young people with cognitive, emotional and behavioural disorders. Despite this surge, many do not seek care from qualified mental health professionals [6].

The first CAMH service was established in late 1990’s at the Sultan Qaboos University Hospital (SQUH) with rudimentary services. More recently, CAMHS has developed a more comprehensive and multidisciplinary approach. Due to the demand, beds are available for urgent admissions, 24 h a day, seven days a week, making it the only psychiatric inpatient service for young people in Oman. It aims to offer comprehensive care for children and young people up to the age of 18 presenting with mental health disorders. The service offers both outpatient and inpatient management, with the latter being a unique service making it mandatory for a caregiver to reside with the young person on the ward during the inpatient stay; such policy has attracted major public appeal, making it the service of choice for inpatient management over the sister institute, Al-Massarah Hospital (AMH), which is the only psychiatry hospital in Oman operated by the Ministry of Health, and has very recently started to offer admissions for young people. In addition, ancillary initiatives such as psychometric services have gained momentum. This is complemented by the necessary social support and by the generation of school reports by psychologists and social workers within the multidisciplinary team. Moreover, in 2017, for the first time, the Ministry of Social Development in Oman launched a 24/7 child protection hotline, which aims to combat child abuse and neglect in the country. The toll-free hotline is a leading national project to protect children in Oman, and works closely with all health care institutes across the country, including CAMHS at SQUH. The unit’s ultimate goal in SQUH is for the multidisciplinary teams to work collaboratively in order to help and assist patients and families to work towards recovery together.

Furthermore, in the last quarter of 2016, a pilot project for adult attention deficit hyperactivity disorder (ADHD) was initiated at SQUH. This was the first and currently the only adult ADHD service in Oman with an ambition to be the centre for research, training, and spreading awareness of adult ADHD. The clinic has been very well received by the service users and their caregivers, as in the past, there was no provision for the management of young people that continue to experience ADHD symptoms into adulthood, with many being discharged from the CAMHS unit to their general practitioners without a comprehensive shared care plan.

Currently, the only two institutes with CAMHS in Oman, SQUH and AMH, offer services to the whole country. Both are located in the capital, Muscat, require many service users to commute long distances, resulting in children missing school and care givers taking the day off work. Therefore, it is due to these current challenges that future perspectives focus on ensuring that more psychiatry trainees pursue careers in child and adolescent psychiatry, with an ambitious vision to develop basic CAMHS in all secondary and tertiary health care providers in Oman.

## Disclosure statement

No potential conflict of interest was reported by the author.
